# The Divergent Metabolomic Landscape of COVID-19 and Community-Acquired Pneumonia

**DOI:** 10.1097/CCE.0000000000001473

**Published:** 2026-09-04

**Authors:** Vassiliki Rapti, Spyros Foutadakis, Nikolaos Kakavoulis, Lamprini Skorda, Evangelos J. Giamarellos-Bourboulis, Garyfallia Poulakou

**Affiliations:** 1 Third Department of Internal Medicine and Laboratory, National and Kapodistrian University of Athens, Medical School, Athens, Greece.; 2 Hellenic Institute for the Study of Sepsis, Athens, Greece.; 3 Second Department of Internal Medicine, Thriasio Elefsis General Hospital, Athens, Greece.; 4 Third Department of Internal Medicine, Korgialeneio-Benakeio Athens General Hospital, Athens, Greece.; 5 Fourth Department of Internal Medicine, National and Kapodistrian University of Athens, Medical School, Athens, Greece.

**Keywords:** arachidonic acid, citric acid cycle, community-acquired pneumonia, COVID-19, metabolomics

## Abstract

**OBJECTIVES:**

To map and compare the serum metabolome of hospitalized patients with COVID-19 or community-acquired pneumonia (CAP).

**DESIGN:**

Observational matched cohort study using an untargeted metabolomics approach.

**SETTING:**

Serum samples were obtained at the time of hospital admission as part of two clinical trials conducted in hospitalized patients.

**PATIENTS:**

A matched cohort design was applied, including patients with COVID-19 and CAP, matched according to age, sex, and Charlson Comorbidity Index. The patient samples were obtained from two clinical studies, namely the suPAR-guided Anakinra Treatment for Validation of the Risk and Management of Respiratory Failure by COVID-19 (SAVE) trial (ClinicalTrials.gov identifier: NCT04357366; European Union Drug Regulating Authorities (EudraCT) number: 2020-001466-11) and the A randomized clinical trial of oral Clarithromycin in Community-acquired pneumonia to attenuatE inflammatory responseS and improve outcomeS (ACCESS trial) (ClinicalTrials.gov identifier: NCT04724044; EudraCT number: 2020-004452-15). The total study population comprised 92 patients.

**INTERVENTIONS:**

None.

**MEASUREMENTS AND MAIN RESULTS:**

A total of 3555 metabolites were detected, and differential analysis recovered more than 70% of the metabolome as altered between conditions. Metabolite pathway analysis highlighted pathways related to the citric acid cycle and arachidonic metabolism as activated in CAP, while COVID-19 was mainly driven by alterations in amino acid metabolism and metabolites related to mitochondrial function.

**CONCLUSIONS:**

Extensive divergence was observed in the metabolomic landscape of patients with COVID-19 or CAP, underscoring the disease-specific metabolic adaptations and providing potential targets for diagnostic and therapeutic development.

**KEY POINTS**

**Question:** Do hospitalized patients with COVID-19 and community-acquired pneumonia (CAP) exhibit distinct serum metabolomic signatures at hospital admission?

**Findings:** In this matched cohort study of 92 hospitalized patients (46 COVID-19, 46 CAP), 3555 serum metabolites were detected, and 2596 differed significantly between groups (73%). CAP was characterized by enrichment of the tricarboxylic acid cycle and arachidonic acid metabolism, whereas COVID-19 showed predominant alterations in amino acid and purine metabolism.

**Meaning:** COVID-19 and CAP are associated with fundamentally distinct host metabolic programs, supporting pathogen-specific immunometabolic adaptation in severe respiratory infection.

Lower respiratory tract infections (LRTIs) continue to represent a substantial global health burden, listed among the leading causes of morbidity and mortality worldwide. Their high prevalence, clinical heterogeneity, and the rising complexity of patient management have maintained LRTIs as a critical focus of infectious diseases research (1). Despite notable advancements in clinical management, the substantial heterogeneity in etiological agents, host responses, and disease trajectories continues to hinder the effective implementation of precision medicine, particularly in identifying high-risk patients and developing tailored therapeutic strategies.

The COVID-19 pandemic marked a milestone, accelerating the application of omics-based technologies, including metabolomics, to enhance diagnostics, elucidate the pathogenesis, assess prognosis, and identify potential therapeutic targets for COVID-19. Although COVID-19 and CAP may present with similar clinical manifestations, their underlying pathophysiological mechanisms differ substantially. Proinflammatory responses prevail in COVID-19 and anti-inflammatory responses in CAP (2). A recent publication from our group has shown that hyperinflammatory responses modulate the metabolomic profile of the host in a far different way than hypoinflammation (3).

Metabolic reprogramming has been recognized as a COVID-19 hallmark, generated by severe acute respiratory syndrome coronavirus 2 (SARS-CoV-2) to facilitate viral replication and modulate immune responses (4). To date, impaired glycolysis, glycerophospholipid remodeling, enriched purine metabolism, disturbed lipid and amino acid metabolism, altered mitochondrial activity, and dysregulated tryptophan/kynurenine pathway are mainly implicated in disease pathogenesis and severity (4). Compared with COVID-19, the metabolic landscape of CAP remains relatively underexplored (5). To the best of our knowledge, direct comparative analyses between CAP and COVID-19 remain scarce.

The present study aims to compare the metabolic profiles of patients with COVID-19 and CAP, providing novel insights into disease-specific mechanisms.

## MATERIALS AND METHODS

Please see the **online supplement** (https://links.lww.com/CCX/B681) for a detailed description of methodologies, including patient cohorts, metabolomics experiments, and bioinformatics analysis.

### Patient Cohorts

A matched cohort of 46 COVID-19 and 46 CAP patients was used for the analysis, with matching based on age, sex, and CCI. Patient samples were derived from two clinical trials: the SAVE trial (ClinicalTrials.gov identifier: NCT04357366; EudraCT number: 2020-001466-11) (6), and the ACCESS trial (ClinicalTrials.gov identifier: NCT04724044; EudraCT number: 2020-004452-15) (7). Patients of both cohorts shared common inclusion criteria and exclusion criteria. More precisely, they were in need of hospitalization and presented with new consolidation on chest radiograph or chest CT. Patients with end-stage malignancy, on chronic intake of corticosteroids and biologicals, and on a Pao_2_/Fio_2_ ratio necessitating mechanical or nonmechanical ventilation were excluded. All participants of the SAVE trial were positive by reverse transcription polymerase chain reaction of the nasopharyngeal exudate for SARS-CoV-2 and were selected to be positive for the biomarker soluble urokinase plasminogen activator receptor (suPAR) (6); all participants of the ACCESS trial were tested negative for SARS-CoV-2 and they selected to meet at least two criteria of the systemic inflammatory response syndrome, to have total sequential organ failure assessment (SOFA) score 2 or more; and to have blood procalcitonin 0.25 ng/mL or more (7).

The present metabolomics study was designed as an exploratory nested analysis within the SAVE and ACCESS trials. Patients with available stored serum samples of sufficient quality and volume for metabolomic analysis were identified from the parent cohorts. From these eligible populations, patients were randomly selected, and a matched study cohort comprising 46 COVID-19 and 46 CAP patients was subsequently assembled according to age, sex, and CCI.

### Metabolomics Measurements

An untargeted metabolomics approach was applied to measure metabolites in patient serum using high-performance liquid chromatography coupled to mass spectrometry (MS) as described in detail in the online supplement. Briefly, upon extracting 10 μL of serum, samples were injected on an iHILIC-(P) Classic high-performance liquid chromatography column (HILICON AB, 100 × 2.1 mm; 5 µm; 200 Å, Sweden) coupled to a high-resolution tandem MS (Orbitrap Exploris 480, Thermo Fisher Scientific, Germany). MS spectra were recorded in polarity switching and MS 1 mode.

In addition to metabolomic profiling, selected immunological parameters generated as part of the predefined analyses of the SAVE and ACCESS studies were retrieved from the central study database. These included interleukin-6 (IL-6) production following a 24-hour ex vivo lipopolysaccharide (LPS) stimulation assay, performed according to the original study protocols (6, 7) and described in detail in the **Supplementary Methods** (https://links.lww.com/CCX/B681). These measurements were not generated specifically for the present metabolomics analysis but were incorporated as complementary indicators of host immune responses.

### Bioinformatics Analysis

The raw metabolomics data were processed by “Compound Discoverer 3.3 SP3” (Thermo Fisher Scientific, Waltham, MA) and manually curated for peak shape and in-source fragments. Different modalities of the MetaboAnalyst software were used for identifying metabolites with differential levels and pathway analysis as further described in the online supplement (https://links.lww.com/CCX/B681) (8).

### Ethics Approval Statement

The present metabolomic analysis was performed using baseline serum samples collected from participants enrolled in two prospective clinical studies conducted in Greece. Patients with COVID-19 were enrolled in the SAVE trial (EudraCT 2020-001466-11; ClinicalTrials.gov NCT04357366), which was approved by the Greek National Ethics Committee (approval 38/20) and the National Organization for Medicines (approval ISO 28/20) (6). Patients with community-acquired pneumonia (CAP) were enrolled in the ACCESS trial (EudraCT 2020-004452-15; ClinicalTrials.gov NCT04724044), which was approved by the Greek National Ethics Committee (approval 122/20) and the National Organization for Medicines (approval IS113/20) (7).

The present study was additionally approved by the Institutional Review Board of Sotiria General Hospital, Athens, Greece, on November 8, 2022 (protocol number 2880/08-11-2022), under the study entitled “Immunosenescence biomarkers in patients with respiratory tract infections: the Case of COVID-19 and community-acquired pneumonia.”

Written informed consent was obtained from all participants or their legally authorized representatives before enrollment. All procedures involving human participants were conducted in accordance with the ethical standards of the responsible institutional and national research committees and with the Declaration of Helsinki and its later amendments.

## RESULTS

### Study Population Characteristics

A total of 92 patients were included in the study, equally divided between the COVID-19 (*n* = 46) and CAP (*n* = 46) cohorts. Baseline demographic characteristics and comorbidity burden were comparable across both groups, including sex distribution (65.2% vs. 56.5% male; *p* = 0.520), mean age (70.9 ± 11.4 vs. 66.4 ± 12.7 yr; *p* = 0.08), and the CCI (3.52 ± 1.68 vs. 2.91 ± 1.74; *p* = 0.091; **Table 1**). Similarly, the prevalence of major comorbid conditions, including diabetes mellitus, congestive heart failure, chronic kidney disease, coronary artery disease, arterial hypertension, and chronic obstructive pulmonary disease, was balanced. Molecularly documented pathogens identified by FilmArray (bioMérieux, Marcy-l’Étoile, France) were recorded when available and are presented in Table 1.

**TABLE 1.** Clinical Characteristics of Study Participants

Variables	COVID-19 (*n* = 46)	Community-acquired pneumonia (*n* = 46)	*p*
Male sex, *n* (%)	30 (65.2)	26 (56.5)	0.520
Age, mean (sd), yr	66.4 (12.7)	70.9 (11.4)	0.077
Charlson Comorbidity Index, mean (sd)	2.91 (1.74)	3.52 (1.68)	0.091
Sequential Organ Failure Assessment, mean (sd)	2.32 (1.33)	4.19 (2.63)	< 0.0001
Acute physiology and chronic health evaluation II, mean (sd)	7.74 (3.55)	12.98 (5.92)	< 0.0001
Medical history, *n* (%)			
Vascular hypertension	25 (54.3)	23 (50.0)	0.855
Diabetes mellitus	10 (21.7)	11 (23.9)	0.834
Dyslipidemia	10 (21.7)	9 (19.6)	0.817
Chronic obstructive pulmonary disease	4 (8.7)	11 (23.9)	0.065
Hypothyroidism	5 (10.9)	7 (15.2)	0.470
Atrial fibrillation	6 (13.0)	5 (10.9)	0.690
Coronary heart disease	5 (10.9)	4 (8.7)	0.628
Chronic heart failure	1 (2.2)	2 (4.3)	0.167
Chronic kidney disease	1 (2.2)	1 (2.2)	1.00
Stroke	2 (4.3)	1 (2.2)	0.167
Laboratory tests			
Lymphocytes (/mm^3^), mean (sd)	1070.82 (501.39)	1293.09 (943.06)	0.167
Procalcitonin, ng/mL, median (quartile 1–quartile 3)	0.19 (0.14–0.50)	1.10 (0.39–1.45)	0.002
C-reactive protein, mg/L, median (quartile 1–quartile 3)	40.2 (13.7–72.0)	202.5 (74.0–284.0)	0.001
Ferritin, ng/mL, mean (sd)	672.92 (697.56)	502.04 (480.59)	0.175
Pao_2_/Fio_2_, mean (sd)	307 (117)	260 (116)	0.056
Molecularly confirmed microorganism by FilmArray of respiratory sample, *n* (%)			
Severe acute respiratory syndrome coronavirus 2	46 (100)	0 (0)	< 0.0001
* Haemophilus influenza*	0 (0)	7 (15.20)	0.012
* Streptococcus pneumonia*	0 (0)	6 (13.04)	0.026
* Haemophilus parainfluenzae*	0 (0)	4 (8.69)	0.116
* Klebsiella pneumonia*	0 (0)	3 (6.52)	0.241
* Staphylococcus aureus*	0 (0)	3 (6.52)	0.241
* Pseudomonas aeruginosa*	0 (0)	2 (4.35)	1.00
* Legionella pneumophila*	0 (0)	1 (2.17)	1.00
Corticosteroid administration	23 (50.00)	14 (30.44)	< 0.001
ICU admission	8 (17.39)	4 (8.69)	0.167
28-d mortality	6 (13.04)	13 (28.26)	0.131
90-d mortality	9 (19.56)	16 (34.78)	0.237

Data are presented as mean (sd), median (interquartile range), or *n* (%), as appropriate. Comparisons between groups were performed using Student *t* test or Mann-Whitney *U* test for continuous variables, and χ^2^ test for categorical variables.

Disease severity indices, as well as inflammatory and biochemical profiles, demonstrated distinct patterns, mostly pronounced in the CAP cohort. Specifically, CAP patients demonstrated higher SOFA scores (4.19 ± 2.63 vs. 2.32 ± 1.33; *p* < 0.0001) and Acute Physiology and Chronic Health Evaluation II scores (12.98 ± 5.92 vs. 7.74 ± 3.55; *p* < 0.0001; Table 1). Furthermore, inflammatory biomarkers, as reflected by median procalcitonin (1.1 vs. 0.195 ng/mL; *p* = 0.002) and C-reactive protein levels (202.5 vs. 40.2 mg/L; *p* = 0.001), were significantly elevated, while greater respiratory impairment was also noted, as indicated by the lower Pao_2_/Fio_2_ ratio (260 vs. 307). In contrast, COVID-19 patients demonstrated a tendency toward higher ferritin (672.92 vs. 502.04) and lower lymphocyte counts (1070.82 vs. 1293), although these differences were not statistically significant. Importantly, LPS-stimulated IL-6 production was markedly elevated in COVID-19 compared with CAP patients (1834.00 vs. 274.48).

Following international therapeutic guidelines, corticosteroids were more frequently administered in COVID-19 patients (50.0% vs. 30.4%; *p* < 0.001).

Finally, even though the ICU admission rate was higher in the COVID-19 group (17.39% vs. 8.69%; *p* = 0.167), no statistical difference was observed in 28-day and 90-day mortality, albeit both outcomes were arithmetically worse in the CAP cohort (13.04% vs. 28.26% and 19.56% vs. 34.78%, respectively).

### The Diverging Metabolomes of Patients With COVID-19 or CAP

Using serum samples from the above matched patient cohorts in untargeted metabolomics experiments, a total of 3555 metabolites with varying levels of confidence in their annotation were detected: 213 metabolites were classified as high-confidence (level 1), 271 as medium-confidence (level 2), and 3071 as low-confidence (level 3). This tiered classification aligns with established standards in untargeted metabolomics and reflects the intrinsic uncertainty associated with metabolite identification on mass spectrometry-based platforms (9).

**Figure 1.**
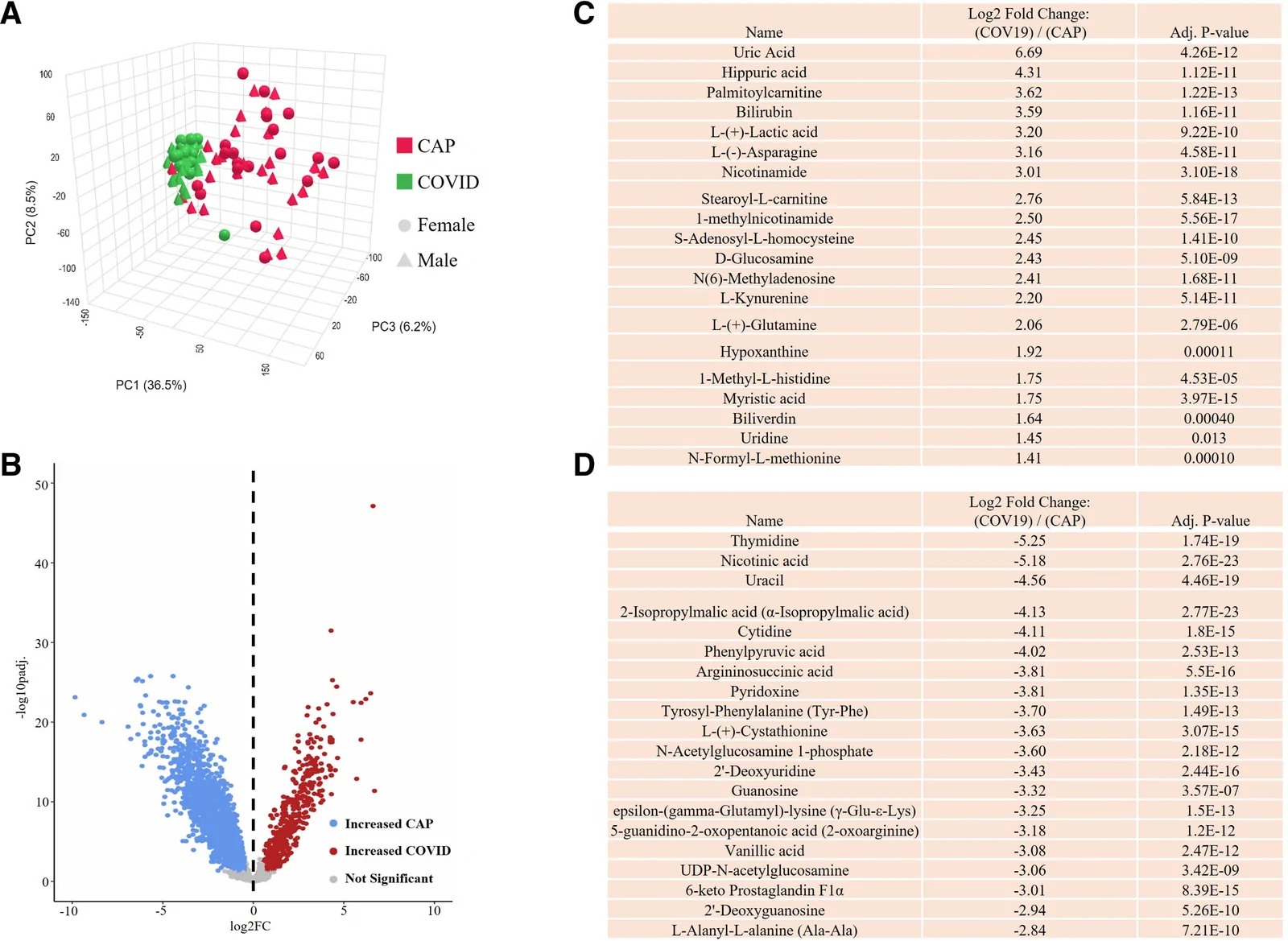
Extensive divergence between the COVID-19 and community-acquired pneumonia (CAP) metabolome. **A**, Principal component analysis plot depicting CAP (shown in *pink*) and COVID-19 samples (shown in *green*) that are further labeled according to sex (male, *triangle*; female, *circle*). COVID-19 samples form a tight cluster that is rather separated from CAP samples that have a more dispersed pattern. **B**, Volcano plot depicting metabolites with increased levels in CAP (2124 metabolites, depicted in *blue*) as well as those with increased levels in COVID-19 samples (472 metabolites, depicted in *red*). The top 20 high-confidence (level 1) metabolites with the highest fold change increases in COVID-19 (**C**) and CAP samples (**D**) are shown. UDP = uridine diphosphate.

To elucidate the metabolic profiles associated with COVID-19 and CAP, principal component analysis was performed. As shown in **Figure 1*A***, COVID-19 samples formed a distinct and tightly clustered group, separated from those of CAP. In contrast, CAP samples exhibited a more dispersed pattern.

Next, we searched for metabolites with differential abundance between the two groups using a threshold of log_2_ fold change greater than 0.5 and an adjusted *p* value of less than 0.05 (**Supplementary Table 1**, https://links.lww.com/CCX/B681). The analysis identified 472 and 2124 metabolites with increased levels in COVID-19 and CAP patients, respectively, as depicted using a Volcano plot (**Fig. 1*B***; **Supplementary Tables 2** and **3**, https://links.lww.com/CCX/B681). Remarkably, more than two-thirds of the metabolites detected (2596/3555; 73%) exhibited significant differences between the groups. We also checked that differential metabolites were spread across all annotation confidence levels. More specifically, 67.6% of confidence level 1 metabolites, 61.9% of confidence level 2 metabolites, and 74.3% of confidence level 3 metabolites had differential abundance between COVID-19 and CAP samples. To rule out that any differences in disease severity could influence the observed metabolome divergence between the two cohorts, we repeated the differential metabolite analysis this time also adding SOFA score as a covariate. This analysis recovered 462 upregulated metabolites in COVID-19 (441 in common with the original analysis) and 2088 in CAP (1898 in common with the original analysis), indicating that any differences in disease severity indices largely do not influence the observed results. Last, given the differential corticosteroid exposure between the two cohorts, additional subgroup analyses were performed following stratification according to corticosteroid administration. Separate comparisons between corticosteroid-treated COVID-19 and CAP patients, as well as between corticosteroid-naive COVID-19 and CAP patients, were conducted. Among high-confidence metabolites, approximately 70% of metabolites upregulated in COVID-19 in the primary analysis remained increased in both subgroup comparisons, whereas 78% of metabolites downregulated in COVID-19 were similarly retained following corticosteroid stratification. A limited number of metabolites related to the citric acid cycle (including dl-isocitric acid and pyruvic acid) and amino acid metabolism (including methionine, threonine, and isoleucine) were differentially altered only among corticosteroid-treated patients.

We went on with the identification of the top 20 high-confidence (group 1) metabolites displaying elevated levels and distinct group-specific patterns (**Fig. 1**, ***C*** and ***D***; **Supplementary Fig. 1**, *A* and *B*, https://links.lww.com/CCX/B681). Regarding COVID-19, uric acid displayed the highest increase (log_2_ fold change = 6.69), followed by hippuric acid (log_2_ fold change = 4.31), palmitoylcarnitine (log_2_ fold change = 3.62), and bilirubin (log_2_ fold change = 3.59; Fig. 1*C*; Supplementary Fig. 1*A*, https://links.lww.com/CCX/B681). Several intermediates involved in energy metabolism and redox regulation were also found elevated, including l-(+)-lactic acid (log_2_ fold change = 3.2), nicotinamide (log_2_ fold change = 3.01), hypoxanthine (log_2_ fold change = 1.92), and biliverdin (log_2_ fold change = 1.64). Last, accumulation of amino acid-related metabolites, such as l-(–)-asparagine (log_2_ fold change = 3.16), *S*-adenosyl-l-homocysteine (log_2_ fold change = 2.45), l-kynurenine (log_2_ fold change = 2.2), 1-methyl-l-histidine (log_2_ fold change = 1.75), and *N*-formyl-l-methionine (log_2_ fold change = 1.41) was also observed. In contrast, nucleotides and nucleotide intermediates (e.g., thymidine [log_2_ fold change = –5.25], nicotinic acid [log_2_ fold change = –5.18], uracil [log_2_ fold change = –4.56], guanoside [log_2_ fold change = –3.32], 2′-deoxyguanosine (log_2_ fold change = –2.94]), as well as catabolic derivatives of amino acids (e.g., phenylpyruvic acid [log_2_ fold change = –4.02], argininosuccinic acid [log_2_ fold change = –3.81]), and key metabolites of the hexosamine biosynthesis pathway (e.g., uridine-*N*-acetylglucosamine [UDP-GlcNAc; log_2_ fold change = –3.06]) were substantially decreased in COVID-19 samples (Fig. 1*D*; Supplementary Fig. 1*B*, https://links.lww.com/CCX/B681).

**Figure 2.**
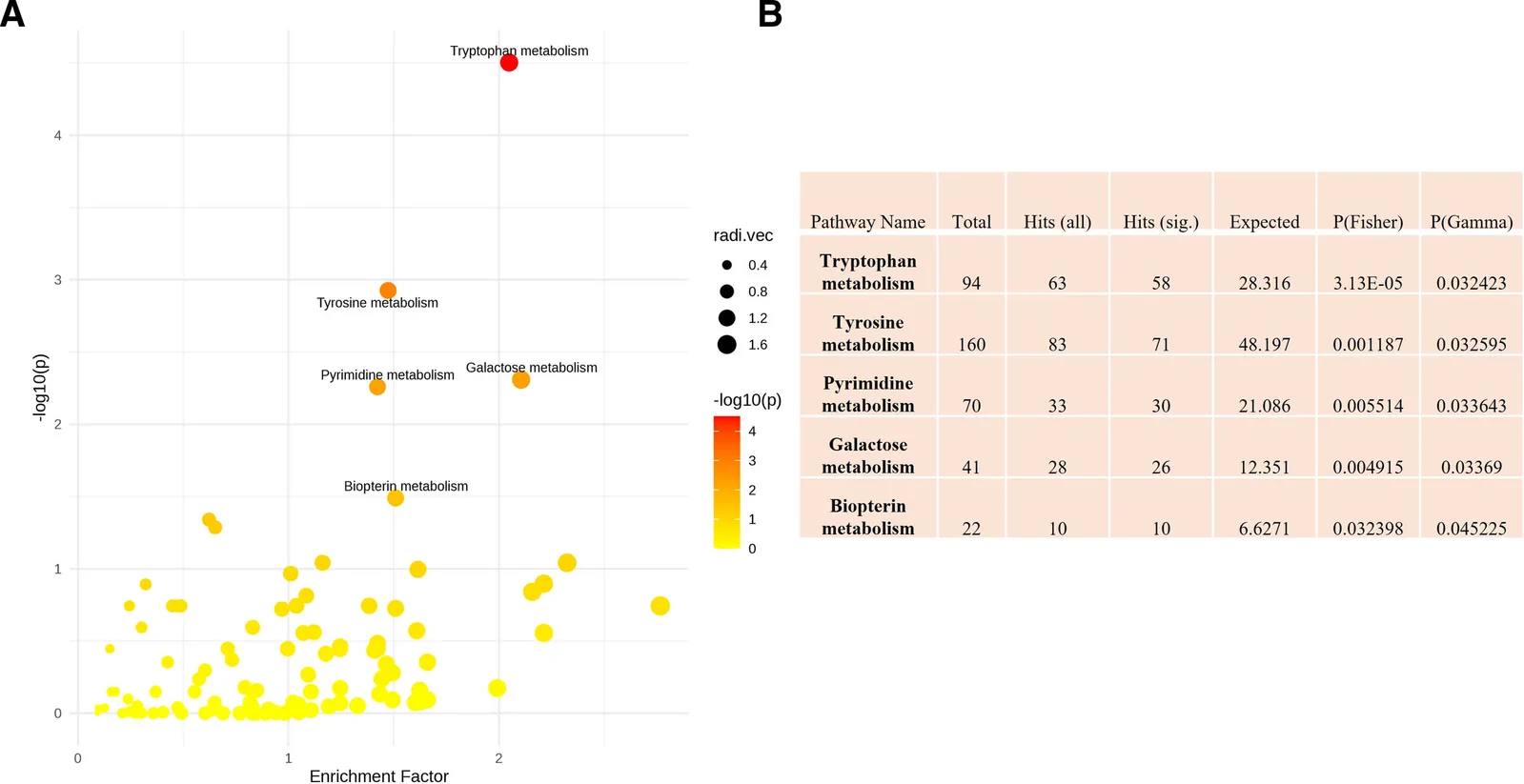
Metabolic pathways enriched among metabolites with altered levels between COVID-19 and community-acquired pneumonia (CAP). **A**, Pathway enrichment analysis using Mummichog. Enriched pathways reaching statistical significance are labeled on the plot and shown in detail in (**B**). Enriched pathways include tryptophan metabolism, tyrosine metabolism, pyrimidine metabolism, galactose metabolism, and biopterin metabolism. Mummichog analysis does not take into account the direction of change in metabolite levels and is used to provide an overview of the enriched metabolic pathways in which differential metabolites partake. The *x*-axis denotes enrichment calculated as the ratio between the number of significant pathway hits and the expected by chance number of hits within that pathway. Enrichment is also indicated by the size of the dots representing each pathway. The *y*-axis indicates the level of statistical significance, which is also illustrated in the *color* of the dots.

We performed an unbiased pathway analysis using the popular Mummichog algorithm to identify enriched metabolic pathways associated with COVID-19 and CAP (10). More specifically, tryptophan, tyrosine, pyrimidine, and galactose metabolism emerged as the most significantly enriched pathways (**Fig. 2**, ***A*** and ***B***; **Supplementary Table 4**, https://links.lww.com/CCX/B681). The Mummichog analysis predicts altered pathways directly from mass spectrometry peaks, thus bypassing the need for upfront metabolite identification and its inherent limitations. Application of Mummichog allowed us to obtain an overview of the altered pathways between COVID-19 and CAP, but since it does not incorporate the directionality of metabolite changes, we complemented this analysis with metabolite set enrichment and Kyoto Encyclopedia of Genes and Genomes (KEGG) pathway analysis separately for upregulated and downregulated metabolites in the following section.

### Critical Metabolic Pathways Associated With COVID-19 or CAP

**Figure 3.**
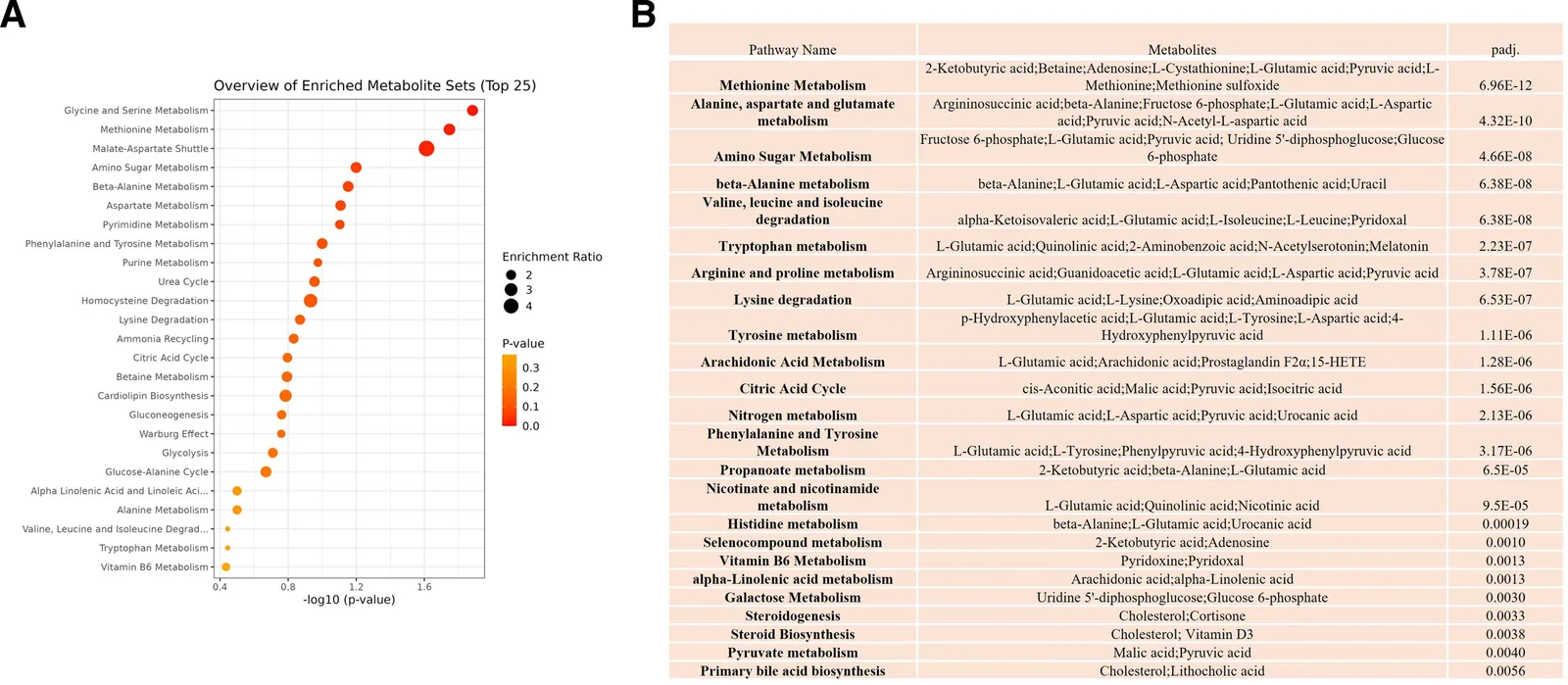
Metabolic sets and pathways enriched among metabolites with increased levels in community-acquired pneumonia (CAP). Metabolite set enrichment analysis (**A**) and Kyoto Encyclopedia of Genes and Genomes (KEGG) pathway analysis (**B**) for high-confidence level 1 metabolites with increased levels in CAP samples, recovered pathways related to amino acid metabolism and the citric acid cycle, among others. HETE = hydroxyeicosatetraenoic acid.

To further delineate disease-specific metabolic alterations, we performed metabolite set enrichment analysis focusing initially on all high-confidence (level 1) metabolites that have increased levels in COVID-19 compared with CAP samples. Among the top 25 enriched pathways, nicotinate and nicotinamide, as well as methionine metabolism, were mostly enriched among others in COVID-19 (**Supplementary Fig. 2***A*, https://links.lww.com/CCX/B681). In contrast, glycine and serine metabolism, methionine metabolism, and the malate-aspartate shuttle exhibited the strongest enrichment in CAP (**Fig. 3*A***).

**Figure 4.**
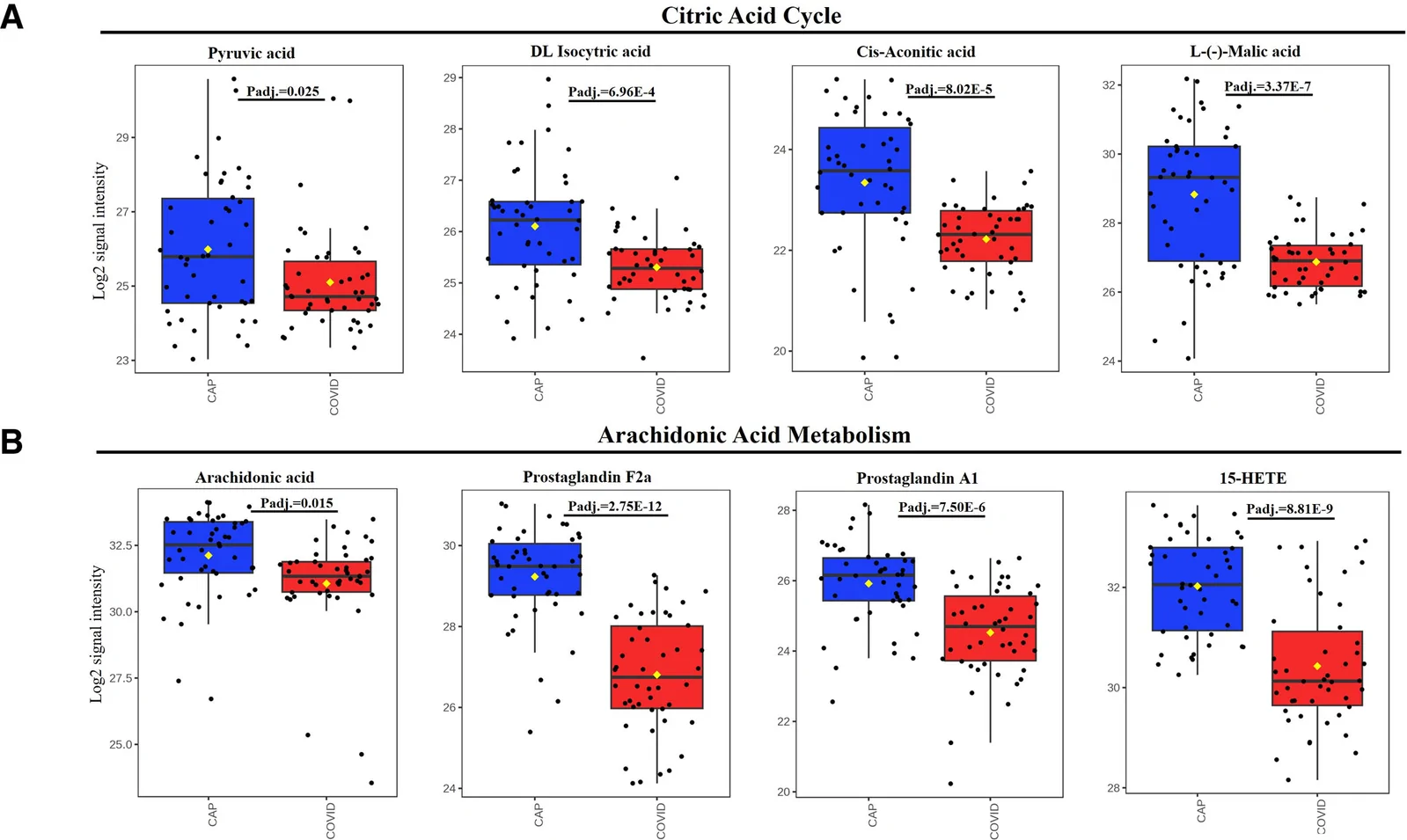
Critical pathways enriched in community-acquired pneumonia (CAP). Several critical metabolites of the citric acid cycle (**A**) and arachidonic acid metabolism (**B**) have increased levels in CAP (depicted in *blue*) compared with COVID-19 (depicted in *red*), indicating heightened activation of these pathways in CAP. HETE = hydroxyeicosatetraenoic acid.

Complementary KEGG pathway analysis largely confirmed the above findings (**Supplementary Figs. 2***B* and **3***B*, https://links.lww.com/CCX/B681 for metabolites upregulated in COVID-19 and CAP, respectively). For example, the characteristic enrichment for metabolites of the citric acid cycle among CAP samples is further presented in **Figure 4*A***. More specifically, four critical members of the citric acid cycle, such as pyruvic acid, isocytric acid, cis-aconitic acid, and malic acid, have increased levels in CAP samples (Fig. 4*A*). The arachidonic acid (AA) metabolism pathway was also recovered as activated in CAP, with characteristic members presented in **Figure 4*B***. These include AA, prostaglandin F2α, prostaglandin A1, and 15-hydroxyeicosatetraenoic acid. It is also important to mention that the identified enriched pathways and metabolic sets are robust to SOFA score-based severity adjustments.

## DISCUSSION

In this study, we conducted a comprehensive metabolomic analysis of COVID-19 and CAP, encompassing over 3500 detected metabolites, and revealing extensive and disease-specific metabolic reprogramming. The present analysis was nested within the prospective SAVE and ACCESS studies, using randomly selected participants with available baseline biospecimens and a predefined matching strategy based on age, sex, and CCI to create two clinically comparable groups for metabolomic analysis. The demographic and clinical characteristics of the selected patients were generally consistent with those reported for the overall SAVE and ACCESS study populations (6, 7).

A key distinction initially emerged in the metabolic profiles: COVID-19 samples formed a tightly clustered group, indicating a higher degree of metabolic homogeneity within the cohort. In contrast, CAP samples presented a more dispersed distribution, likely reflecting the diversity of causative pathogens, variability in disease severity, and heterogeneity in host immune responses. Nevertheless, this observation should be interpreted with caution, as the tighter clustering observed in COVID-19 samples may also partly reflect the presence of a single dominant pathogen (SARS-CoV-2), whereas the CAP cohort included multiple bacterial etiologies associated with greater biological heterogeneity. Furthermore, nearly 70% of the identified metabolites displayed differential abundance between the two groups, underscoring the breadth of metabolic divergence. These findings highlight the fundamentally different biological mechanisms underlying COVID-19 and CAP pathogenesis.

Collectively, the pathway-level alterations identified in this study point to a distinct metabolic reprogramming in COVID-19 compared with CAP. In line with the existing literature (4), COVID-19 pathogenesis was characterized by alterations in purine, amino acid, and tryptophan metabolism, as well as pathways related to mitochondrial function. Importantly, these findings were observed in a COVID-19 population at great risk for adverse outcomes, as reflected by elevated suPAR levels (6). Notably, several pathways, including methionine and tryptophan metabolism, were enriched in both COVID-19 and CAP, likely reflecting shared host responses to severe respiratory infection, such as inflammation, oxidative stress, and immune activation. However, the metabolites contributing to pathway enrichment and their relative abundance patterns differed substantially between the two conditions, supporting the presence of distinct immunometabolic programs despite partial overlap in pathway signatures. Previous studies have similarly implicated disruptions in purine, tryptophan, and amino acid metabolism in immune dysregulation during severe SARS-CoV-2 infection (11–14).

The last major distinction was observed at the level of core metabolic pathways, specifically within the citric acid cycle and AA metabolism. Beyond bioenergetic disruption, the accumulation of selected citric acid cycle intermediates and pyruvate in CAP may be compatible with broader immunometabolic adaptations previously described in inflammatory states, including features resembling a Warburg-like response; however, dedicated functional studies would be required to confirm this interpretation (15). For instance, itaconate, derived from cis-aconitic acid in LPS-activated macrophages, exerts its immunosuppressive properties mainly through the regulation of hypoxia-induced factor (HIF)-1α-IL-1β axis, activation of the anti-inflammatory transcription factor nuclear factor erythroid 2-related factor 2 (nuclear factor erythroid 2 like 2) followed by the downstream expression of inflammatory genes, and modulation of type I interferon signaling as part of a negative feedback loop (16, 17). While alpha-ketoglutaric has been implicated in the inhibition of proinflammatory pathways, such as nuclear factor kappa B and HIF-1α signaling, as well as epigenetic regulation through histone and DNA demethylation (16, 18), elevated levels of pyruvic acid in the monocytes of patients suffering from CAP may present a compensatory mechanism to support residual cytokine production despite an overall hyporesponsive immune state (16, 19). Furthermore, our previous work has shown analogous metabolic defects in patients with sepsis, with deregulation of glycolysis and oxidative metabolism. Importantly, those were reverted following administration of recombinant interferon-γ, offering a putative therapeutic for such metabolic defects (20). On the contrary, a shift toward anaerobic glycolysis and lipid-derived energy production was characteristic in the COVID-19 cohort, as already mentioned. Of note, severe COVID-19 has been associated with down-regulation of glycolytic and citric acid cycle activity, accompanied by enhanced activation of immune signaling pathways such as IL-6, tumor necrosis factor, and T cell receptor pathways (21).

In our study, the activated AA metabolic cascade was predominantly evident in CAP samples. This finding aligns with prior lipidomics-based research on CAP-associated sepsis, which revealed extensive time-dependent alterations in the lipidome correlated with disease severity and inflammatory markers (22). Furthermore, in another study exploring the lipidome profiles of patients with severe COVID-19 and CAP presenting signs of acute respiratory failure, phosphatidylcholine, the principal reservoir of AA, was significantly reduced in COVID-19 (23).

So far, a limited number of studies have directly compared the metabolomic landscapes of COVID-19 and CAP. A recent study used ultra-performance liquid chromatography–tandem mass spectrometry (UPLC-MS/MS) and reported alterations in pathways such as AA metabolism, purine metabolism, as well as lysine degradation, beta-alanine, histidine, taurine, and hypotaurine metabolism (24). Nevertheless, a limited number of differential metabolites was reported, and the total coverage was only a few hundred metabolites. Another study by Saballs et al (25) applied NMR to investigate the metabolome of hospitalized patients suffering from either community-acquired or COVID-19 pneumonia and also compared it to healthy controls. Their methodology examines a very limited number of metabolites, mainly amino acids such as alanine, glycine, and creatine, that were found increased in patient samples compared with healthy controls. Regarding valine and alanine levels, their results are incongruent with ours, as they found increased levels in COVID-19 samples compared with CAP. This highlights the need for further, larger studies to clarify putative pathogen-specific effects (25).

Despite the strengths of this study, some limitations should be acknowledged. First, the relatively modest sample size and the exploratory design may limit the generalizability of the findings to other populations and clinical settings. Second, the untargeted metabolomics approach inherently yielded a substantial proportion of low-confidence metabolite annotations, while the analysis was based on a single baseline sampling time point, precluding assessment of dynamic metabolic changes during disease evolution. Third, differences in corticosteroid exposure, together with the fact that the COVID-19 and CAP cohorts were derived from two independent prospective clinical trials with distinct enrollment criteria, may have contributed to the observed metabolomic differences. In addition, detailed information regarding the exact timing of corticosteroid administration relative to baseline serum sample collection was not available for the present analysis, precluding assessment of the potential acute effects of steroid exposure on the metabolomic profiles. Although matching and multivariable adjustment, including incorporation of SOFA score as a covariate, reduced baseline imbalances and the principal metabolomic signatures were largely preserved after corticosteroid-stratified and SOFA-adjusted analyses, residual confounding related to treatment exposure, trial of origin, and disease severity cannot be entirely excluded. Therefore, the observed metabolomic differences should be interpreted as exploratory associations between disease category and the serum metabolome rather than definitive pathogen-specific metabolic signatures. Finally, functional validation experiments were not performed and will be important for confirming the biological relevance of the identified pathways.

## CONCLUSIONS

Τo conclude, this study emphasizes marked divergence in the serum metabolomic landscape of hospitalized patients with COVID-19 and CAP. Pathway-level analyses revealed distinct metabolic signatures associated, highlighting differential remodeling of energy, amino acid, and immune-related metabolic pathways. Together, these findings portray the complexity of host metabolic responses in severe respiratory infections and emphasize the value of pathway-level analyses for delineating disease-specific metabolic remodeling.

Drs. Giamarellos-Bourboulis and Poulakou conceived, designed, and funded the study. Primary data analysis was performed by Drs. Foutadakis and Rapti. The first draft was prepared by Drs. Rapti and Foutadakis, and all authors contributed to previous versions of the article. All authors approved the final article.

## Supplementary Material
