# Supplementary material for: The Divergent Metabolomic Landscape of COVID-19 and Community-Acquired Pneumonia
Source: Crit Care Explor. 2026 Sep 4;8(9):e1473. doi: 10.1097/CCE.0000000000001473 (PMC13549596; doi:10.1097/CCE.0000000000001473)
Supplement: Supplementary file 1 [file cc9-8-e1473-s001.pdf]

ONLINE DATA SUPPLEMENT

The divergent metabolomic landscape of COVID-19 and community-acquired pneumonia

Vassiliki Rapti, MD, MSc[1]; Spyros Foutadakis, PhD [2]; Nikolaos Kakavoulis, MD, MSc[3]; Lamprini Skorda, MD, PhD[4] ; Evangelos J Giamarellos-Bourboulis, MD, PhD, FISAC[2,5]; Garyfallia Poulakou, MD, PhD[1]

SUPPLEMENTAL METHODS

Patient Cohorts

A matched cohort of 46 COVID-19 and 46 CAP patients was used for the metabolomics analysis, with matching based on age, sex, and Charlson Comorbidity Index (CCI). Patient samples were derived from two clinical trials: the SAVE (suPAR-guided Anakinra treatment for Validation of the risk and Early management of SRF by COVID-19; ClinicalTrials.gov identifier: NCT04357366; EudraCT number:2020-005828-11) (1) and the ACCESS trial (Clarithromycin for Early Anti-inflammatory Responses in Community-Acquired Pneumonia; ClinicalTrials.gov identifier: NCT04724044; EudraCT number: 2020-004452-15) (2). For the purposes of the present metabolomic analysis, only baseline samples collected on the first day of study enrollment were considered. To ensure that the metabolic profiles reflected the untreated state, samples were obtained prior to the administration of either the study drug or placebo, and only patients who fully adhered to all protocol-defined procedures were included in the analysis.

Briefly, the COVID-19 cohort consisted of hospitalized adult patients with molecularly confirmed SARS-CoV-2 infection detected in nasopharyngeal secretions, accompanied by radiological evidence of lower respiratory tract infection (LRTI). Eligibility required a plasma concentration of soluble urokinase plasminogen activator receptor (suPAR) ≥6 ng/mL, as determined using the suPARnostic® Quick Triage kit (Virogates S/A, Blokken 45, 3460 Birkerød, Denmark). At the time of study initiation, dexamethasone had not yet been incorporated into the standard of care for COVID-19, and remdesivir had not yet received regulatory authorization. Patients were excluded if they met any of the following conditions: stage IV malignancy; any do not resuscitate decision; ratio of partial oxygen pressure to the fraction of inspired oxygen (PaO_2/FiO_2) ratio <150 mmHg; requirement for either invasive mechanical ventilation (MV) or non-invasive ventilation (NIV) with positive pressure support; diagnosis of a primary immunodeficiency; neutropenia defined as an absolute neutrophil count <1500/mm^3; administration of systemic corticosteroids at a daily dose ≥0.4

mg/kg prednisone (or equivalent) within the preceding 15 days; receipt of anti-cytokine biological therapy within the prior month; or ongoing pregnancy or lactation. In the subsequent months, however, dexamethasone was increasingly administered as part of routine clinical practice, whereas remdesivir was administered only to a comparatively limited number of patients (1).

The CAP cohort included hospitalized adult patients of either sex with radiologically confirmed CAP. Eligible patients were required to present with at least two characteristic respiratory symptoms (cough, purulent sputum production, dyspnea, or pleuritic chest pain), fulfill at least two systemic inflammatory response syndrome (SIRS) criteria, have a baseline Sequential Organ Failure Assessment (SOFA) score ≥2, and demonstrate a serum procalcitonin concentration ≥0.25 ng/ml. For women of childbearing potential, eligibility additionally required documented non-pregnancy, confirmed by a urine pregnancy test prior to enrollment, and assurance of continued use of at least one effective contraceptive method for the duration of the study (e.g., oral contraceptives or barrier protection). Exclusion criteria comprised the following: age <18 years; refusal or inability to provide written informed consent; prior intake of any macrolide for the current episode of CAP under investigation; administration of systemic corticosteroids at a dose exceeding 0.4 mg/kg/day of prednisone (or equivalent) within the preceding 15 days; neutropenia, defined as an absolute neutrophil count <1,000/mm^3; known infection with human immunodeficiency virus (HIV); receipt of chronic anti-cytokine therapy (e.g., anti-TNF antibodies for rheumatoid arthritis); documented infection with SARS-CoV-2; hospitalization for more than 2 days within the past 90 days; a corrected QT interval (QTc) ≥500 msec on resting electrocardiogram or history of congenital long QT syndrome; concomitant treatment with HMG-CoA reductase inhibitors (statins) extensively metabolized by CYP3A4 (i.e., lovastatin or simvastatin); or any additional contraindication to the investigational drug. Female patients of childbearing potential were excluded in the presence of pregnancy or lactation, with non-pregnancy confirmed by a urine pregnancy test prior to enrollment. All enrolled patients received β-lactam monotherapy or β-lactam/β-lactamase inhibitor combinations as standard-of-care treatment (2).

In both trials, written informed consent was obtained from all patients prior to any screening procedures; in cases where patients lacked decision-making capacity, consent was instead provided by a first-degree relative (1,2).

Untargeted metabolomics

The measurement and annotation of metabolites was performed by the metabolomics facility at Vienna BioCenter (Austria, Europe). A starting 10 µL of serum was extracted with 150 µL methanol (Fisher Chemical, OPTIMA®)-ethanol (Supelco, EMSURE®) solvent (MeOH:EtOH:H_2O; 4:1:1 v/v), vortexed, and chilled at -20 °C for one hour. Afterwards, the samples were centrifuged at 4°C and 20,000 x g for 10 min. Then the supernatant was diluted with acetonitrile-CAN (HiPerSolv CHROMANORM) (1:1) and the samples were subjected to analysis with liquid chromatography-mass spectrometry (LC-MS). 1.7 µL of each diluted sample was pooled and served as a quality control (QC) sample. Samples were randomly injected on an iHILIC®-(P) Classic HPLC column (HILICON AB, 100 x 2.1 mm; 5 µm; 200 Å, Sweden) equipped with a guard column (HILICON AB, 20 x 2.1 mm; 5 µm; 200 Å, Sweden). The flow rate was adjusted to 100 µl/min delivered through an Ultimate 3000 HPLC system (Thermo Fisher Scientific, Germany). The stepwise gradient has a total run time of 35 min, starts at 90% A (ACN) and takes 21 min to 60% B (25 mM ammonium bicarbonate), followed by a 5 min hold at 80% B and a subsequent equilibration phase at 90%. The LC was coupled to a high-resolution tandem MS (Orbitrap Exploris 480, Thermo Fisher Scientific, Germany). Ionization potential was set to +3.4/-2.8 kV, the sheet gas flow to 18, and an auxiliary gas flow of 7 was selected. MS spectra were recorded in polarity switching and MS^1 mode. EASY-IC lock mass correction was selected with the scan-to-scan mode. Upon equilibrating the system by injecting the QC sample 7 times, repeatedly ten random samples derived from both groups were flanked by an extraction blank and a QC sample for background labeling and data normalization, respectively. QC samples as well as selected samples of each group were also measured in data dependent acquisition to obtain fragmentation spectra for metabolite identification. The obtained data set was processed by

“Compound Discoverer 3.3 SP3” (Thermo Fisher Scientific) and manually curated for peak shape and in-source fragments. The features were annotated through searching against an in-house mass list database generated using authentic standard solutions. Additionally, the mzCloud database was searched for fragment matching and ChemSpider hits were obtained using BioCyc, the human metabolome database, KEGG, and the serum metabolome database.

Differential abundance analysis

Differential abundance analysis was implemented using the statistical analysis (metadata table) module from within the MetaboAnalyst version 6.0 platform (3). More specifically, log2 normalized metabolite peak intensities and a metadata table containing age, sex and CCI as variables were given as input, without any further normalization or scaling. A linear model using limma was applied to perform significance testing, also correcting for the above metadata (4). As significant were considered those metabolites with log2foldchange>0.5 and FDR-corrected p-value<0.05.

Visualization tools

Heatmaps were constructed using Morpheus (5). A matrix containing the metabolite peak intensities for each sample, together with a metadata table containing age, sex and CCI as variables, was provided as input. The matrix was log2 transformed, followed by z-score normalization. Volcano plots were prepared using the Galaxy suite (6). Peak intensities for selected metabolites were visualized using the MetaboAnalyst built-in boxplot functionality. Finally, PCA plots were constructed using the synchronized 3D plots from the iPCA functionality within MetaboAnalyst.

Pathway Enrichment Analysis

Pathway enrichment analysis was implemented in MetaboAnalyst, version 6.0 (3), using the Functional Analysis module, which invokes the Mummichog algorithm (7). Given the inherent challenges in unambiguous metabolite identification/assignment, Mummichog

bypasses the need for upfront metabolite identification and predicts enriched pathways directly from spectral feature tables. More specifically, a file containing the ion's m/z features, p-value and whether it was identified in positive or negative mode was given as input to the program. Moreover, the analysis parameters included ion mode: mixed mode; mass tolerance: 3 ppm; retention time: not present; ranked by p-value and Enforce Primary Ions. All detected metabolites were used as an input for the Mummichog analysis, with a p-value significance cutoff of 10^{-5}. Those metabolites that did not reach this significance were used as the background. The Homo sapiens Molecular Feature Network library was used and analysis was restricted to pathways with at least three entries (7). Finally, metabolic pathways were reported as significant when the Mummichog-derived p-value of the Fisher statistical test was below 0.05.

Metabolite set analysis

Metabolite set analysis was performed using MetaboAnalyst, version 6.0, with the Enrichment Analysis module (3). A list of HMDB IDs representing high-confidence (level 1) metabolites with increased levels in either the CAP or COVID cohort was separately given as input. Moreover, the SMPDB library of metabolite sets was used (E8), restricting the analysis to those sets containing at least two entries and having the totality of compounds in HMDB as the reference metabolome. The top 25 metabolite sets were plotted, irrespective of their p-values.

Metabolite pathway analysis

Metabolite pathway analysis was performed with RAMP (9). A list of HMDB IDs representing high-confidence (level 1) metabolites with increased levels in either the CAP or COVID cohort was separately provided as input using blood tissue as the background option. The focus was only on results derived from the KEGG database using a FDR-corrected $p<0.05$ as a cutoff.

Ex vivo PBMC stimulation assay and IL-6 measurements

IL-6 measurements following 24-hour ex vivo lipopolysaccharide (LPS) stimulation were retrieved from the central databases of the prospective SAVE and ACCESS studies. These measurements were generated as part of the predefined immunological investigations of the original study protocols (1,2), and were not performed specifically for the present metabolomics analysis but were incorporated as complementary indicators of host immune responses.

Peripheral blood mononuclear cells (PBMCs) were isolated from whole blood by Ficoll density-gradient centrifugation, washed with phosphate-buffered saline, and cultured in RPMI 1640 medium under standard cell culture conditions. PBMCs were incubated for 24 hours in the absence or presence of 10 ng/mL *Escherichia coli* O55:B5 lipopolysaccharide (LPS) at 37°C and 5% CO_2. Following incubation, cell culture supernatants were collected, and interleukin-6 (IL-6) concentrations were determined by enzyme-linked immunosorbent assay (ELISA). The IL-6 values reported in the present study correspond to cytokine production after 24-hour ex vivo LPS stimulation. Detailed methodological information regarding the immunological assays is available in the original SAVE and ACCESS study publications (1,2).

REFERENCES

1. Kyriazopoulou E, Panagopoulos P, Metallidis S, et al: An open label trial of anakinra to prevent respiratory failure in COVID-19. *Elife* 2021; 10:e66125

2. Giamarellos-Bourboulis EJ, Siampanos A, Bolanou A, et al: Clarithromycin for early anti-inflammatory responses in community-acquired pneumonia in Greece (ACCESS): a randomised, double-blind, placebo-controlled trial. *Lancet Respir Med* 2024; 12:294-304

3. Pang Z, Lu Y, Zhou G, et al: MetaboAnalyst 6.0: towards a unified platform for metabolomics data processing, analysis and interpretation. *Nucleic Acids Res* 2024; 52:W398-W406

4. Ritchie ME, Phipson B, Wu D, et al. limma powers differential expression analyses for RNA-sequencing and microarray studies. Nucleic *Acids Res* 2015; 43:e47

5. MORPHEUS: Versatile matrix visualization and analysis software. Available at: https://software.broadinstitute.org/morpheus/. Accessed August 1, 2025

6. Galaxy Community: The Galaxy platform for accessible, reproducible, and collaborative data analyses: 2024 update. *Nucleic Acids Res* 2024; 52:W83-W94

7. Li S, Park Y, Duraisingham S, et al: Predicting network activity from high throughput metabolomics. *PLoS Comput Biol* 2013; 9:e1003123

8. Jewison T, Su Y, Disfany FM, et al: SMPDB 2.0: big improvements to the Small Molecule Pathway Database. *Nucleic Acids Res* 2014; 42:D478-84

9. Braisted J, Patt A, Tindall C, et al: RaMP-DB 2.0: a renovated knowledgebase for deriving biological and chemical insight from metabolites, proteins, and genes. *Bioinformatics* 2023; 39:btac726

Supplementary Figures

Supplementary Figure 1:

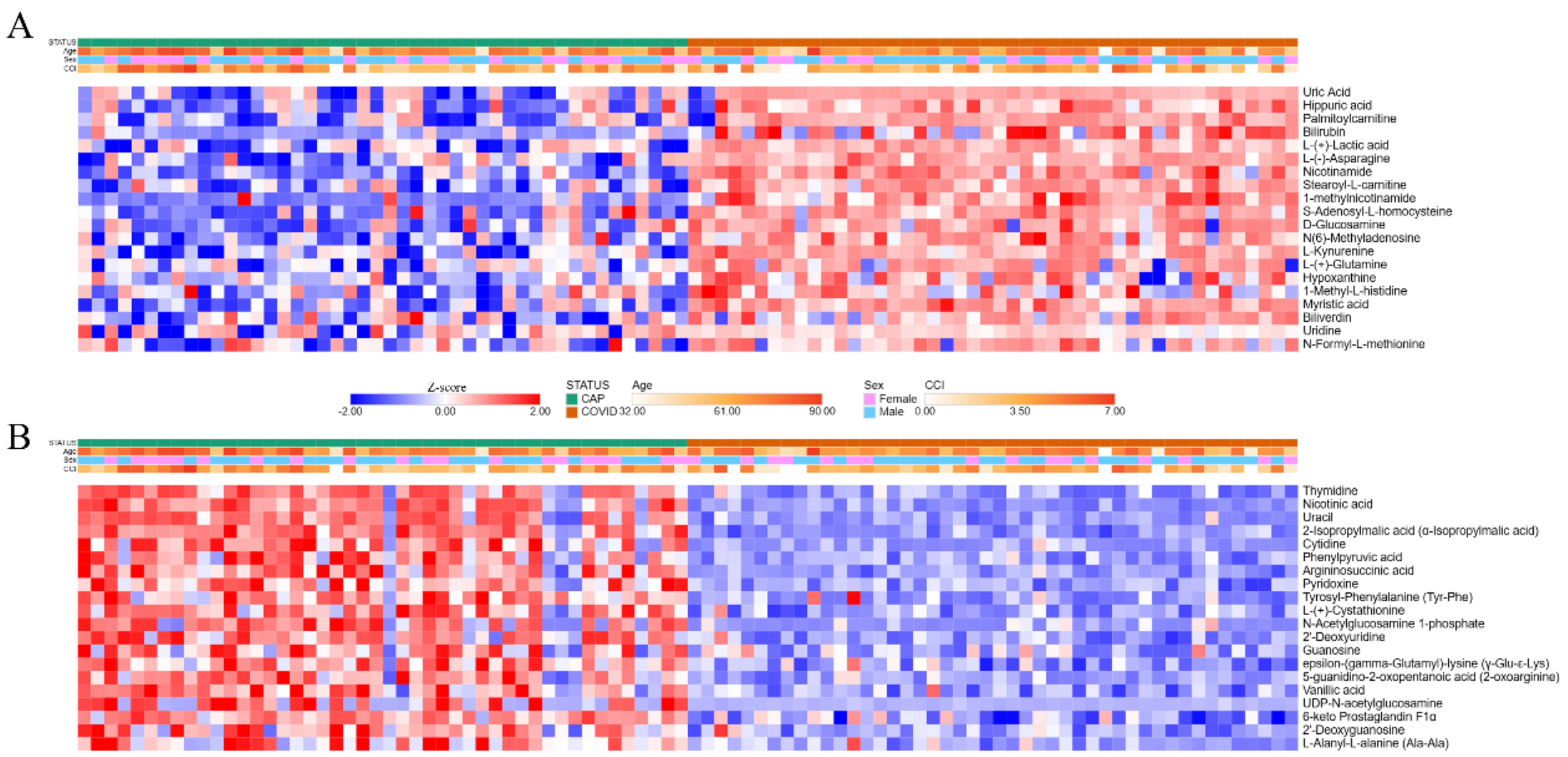

Supplementary Figure 1: Heatmaps illustrate the top enriched metabolites in COVID-19 and CAP samples. Shown are heatmaps depicting metabolite levels across samples for the top20 metabolites that have increased levels in COVID-19 (A) and CAP (B). CAP samples are shown in green on the left half of the heatmaps, while COVID-19 samples are on the right side, colored in brown. Age, Sex and CCI metadata are also shown for all samples.

Supplementary Figure 2:

A

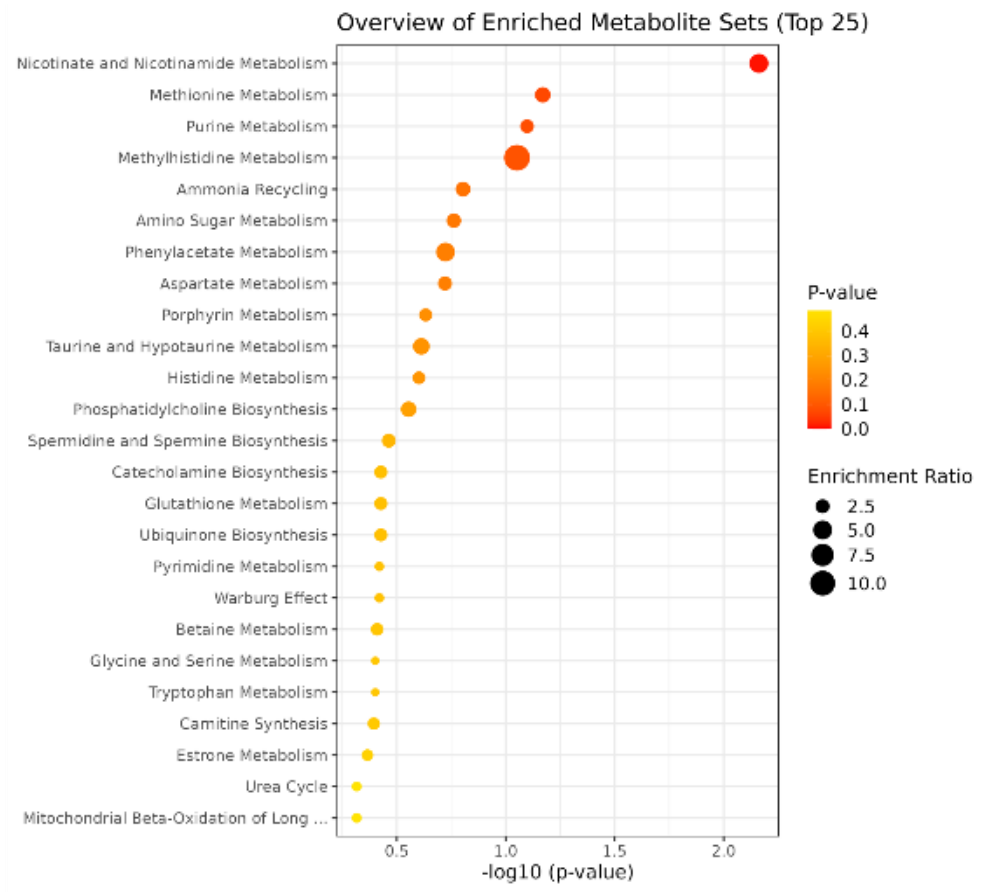

B

Pathway Name	Metabolites	padj.
**Nicotinate and nicotinamide metabolism**	1-Methylnicotinamide;S-Adenosylhomocysteine;Niacinamide;L-Glutamine	2.08E-07
**Amino Sugar Metabolism**	D-Fructose;Glucosamine;L-Glutamine	1.3E-05
**Methionine Metabolism**	Sarcosine;S-Adenosylhomocysteine;5'-Methylthioadenosine	6.9E-05
**Nitrogen metabolism**	L-Asparagine;L-Glutamine	0.00057
**Porphyrin Metabolism**	Bilirubin;Biliverdin	0.00079
**Histidine metabolism**	1-Methylhistidine;S-Adenosylhomocysteine	0.00089
**Tryptophan metabolism**	L-Kynurenine;S-Adenosylhomocysteine	0.0012
**Arginine and proline metabolism**	S-Adenosylhomocysteine;L-Glutamine	0.0015
**Alanine, aspartate and glutamate metabolism**	L-Asparagine;L-Glutamine	0.0020

Supplementary Figure 2: Metabolic sets and pathways enriched among metabolites with increased levels in COVID-19. Metabolite set enrichment analysis (A) and KEGG pathway analysis (B) for high confidence level 1 metabolites with increased levels in COVID-19 samples identified pathways related to amino acid and purine metabolism, among others.
